# Authors’ Response to Peer Reviews of “Monte Carlo Dose Estimation of Absorbed Dose to the Hematopoietic Stem Cell Layer of the Bone Marrow Assuming Nonuniform Distribution Around the Vascular Endothelium of the Bone Marrow: Simulation and Analysis Study”

**DOI:** 10.2196/77812

**Published:** 2025-07-16

**Authors:** Noriko Kobayashi

**Affiliations:** 1 Individual Researcher, 4-16-18-1F Hamadayama Suginami-ku, Tokyo, 16380065, Japan, 81 03 5929 7201

**Keywords:** stem cells, radiation, bone marrow, nuclides, noble gases

*This is the authors’ response to peer-review reports for “Monte Carlo Dose Estimation of Absorbed Dose to the Hematopoietic Stem Cell Layer of the Bone Marrow Assuming Nonuniform Distribution Around the Vascular Endothelium of the Bone Marrow: Simulation and Analysis Study.”*

## Round 1 Review

### Reviewer T [1]

#### General Comments

*In this study [* *2* *], a geometric model of trabecular bone and bone marrow tissue was constructed at the micrometer scale, assuming that the hematopoietic stem cells layer was localized in the perivascular hematopoietic stem cell layer of the sinusoids. The absorbed doses of the stem cell layer from blood and trabecular bone sources were then estimated for selected β nuclides, α nuclides, and noble gases and compared with the specific absorbed fractions (SAFs) values of International Commission on Radiological Protection (ICRP) 60 and 103. It was concluded that the absorbed doses from the bone marrow and blood sources were greater than those from trabecular bone sources for α nuclides, and the total absorbed dose was lower than that estimated from the current ICRP models.*

#### Specific Comments

*The results were tabulated; however, it was not clear how the comparison between the Particle and Heavy Ion Transport System, ICRP 60, and ICRP 103 was performed, what test was used, and the level of significance. Even in Table 7 that summarizes the results, this is not clear.*

**Response:** Because the energy and spectrum of each individual nuclide are completely different, it is not possible to calculate and compare with *P* values from data on different nuclides. In addition, because rare gases and radon are not currently being evaluated, they are not comparable.

*The abbreviations throughout the article need to be identified. It is recommended to add an abbreviation section to the article.*

**Response:** Abbreviations such as “TB” (trabecular bone) and “RBM” (red bone marrow) have been modified to match the terminology used by the ICRP.

*The abstract section is better structured as Background, Objectives, Methods, Results, and Conclusion.*

**Response:** Revised.

*In the abstract section, the authors mentioned that the absorbed doses to the bone marrow obtained from the model calculations were not significantly different from ICRP 60 and ICRP 103 for β nuclides. Still, they were much lower than previously estimated for α nuclides. Going through the study, it was not clear how this significant difference was assessed. Please revise and clarify.*

**Response:** For each nuclide, calculations are performed using Monte Carlo simulation until the statistical error is sufficiently low.

*The abbreviation “SAFs” in the keyword section and the last paragraph of the Introduction section should be identified as the “specific absorbed fractions.”*

**Response:** Revised.

*The abbreviation “PHITS” in the keyword section and the first line of the fourth page should be identified as ”Particle and Heavy Ion Transport System.”*

**Response:** Revised.

*The abbreviation “keV” in the last line of the second paragraph of the seventh page should be identified as “kilo electron-volt.”*

**Response:** Revised.

*In the last line of the second paragraph of the seventh page, please identify “Bremsstrahlung” as a type of X-radiation emitted by charged particles when they collide or are near an atomic nucleus.*

**Response:** Revised.

*The abbreviation “EGS” in the last line of the second paragraph of the seventh page should be identified as “Electron Gamma Shower.”*

**Response:** Revised.

*The abbreviation “Bq” in the first line of the last paragraph of the seventh page should be identified as “The International System of Units (SI) unit of radionuclide activity is the becquerel (Bq); 1 Bq = 1 transformation/second.”*

**Response:** Revised.

*First line, page 10: Please correct “131” to “131I.”*

**Response:** Revised.

*Page 16, Discussion section, last line of the first paragraph: The authors mentioned that the number of decays in each compartment changed significantly; how did the authors assess this significant change and conclude it? Please explain the tests used for comparison.*

**Response:** The word was not used to mean statistically significant but rather to mean that the number of decay has changed significantly.

*Page 16, Discussion section, eighth line of the second paragraph: Please revise “ICRP133 SAF” (mentioned in the Results section as “ICRP103 SAF”).*

**Response:** Revised.

*Page 17, last line of the first paragraph: “Sakota et al” should be corrected to “Sakoda et al.”*

### Reviewer V [3]

#### Abstract Section

*The manuscript’s abstract begins with a statement about hematopoietic stem cells’ proximity to sinusoidal capillaries but does not clarify why this spatial distribution is relevant for radiation dosimetry until later in the text. A clearer explanation linking the hematopoietic stem cell location with the dosimetric model limitations would better engage readers unfamiliar with the topic.*

**Response:** The following sentence has been added to the abstract: “If the location of the hematopoietic stem cell layer differs from previous assumptions, it will be necessary to re-evaluate the dose, particularly for alpha rays with a short range.”

*Some sentences are overly complex, especially in the Introduction and Conclusion. Simplifying the language or splitting ideas across multiple sentences could improve readability.*

**Response:** I’ve divided the sentences to improve readability and clarity, as shown in the revised version.

*The abstract lacks methodological detail regarding how the model calculations were performed. Including brief specifics about the model’s approach, particularly the role of computed tomography imaging if applicable, would improve transparency and give context to the reported findings.*

**Response:** Revised.

*The results comparing the absorbed doses for α and β nuclides are presented with limited interpretation. The abstract states that doses for β nuclides were similar to ICRP estimates, while those for α nuclides were much lower, yet there is no explanation for the potential reasons behind these differences. Offering a brief discussion or hypothesis, even speculative, would enrich the reader’s understanding.*

**Response:** The following sentence was added: “Particularly, in the case of alpha-emitting nuclides with a short range, the alpha particles may not reach the vascular endothelium from the bone source.”

#### Introduction Section

*The Introduction could benefit from a clearer structure. Currently, it presents information about various models and dosimetric approaches in a somewhat fragmented manner.*

**Response:** Revised.

*Certain technical terms such as “surrogate target,” “trabecular bone surface,” “endosteum,” and “standard absorbed fraction” may benefit from concise explanations or definitions. For instance, briefly defining “surrogate target” would help those unfamiliar with dosimetry or radiobiology terminology.*

**Response:** Added explanations of terms such as SAF and endosteal layer in the text.

#### Method Section

*The study uses an intricate geometric model based on JM-103 data, Particle and Heavy Ion Transport System software, and Japan Atomic Energy Agency guidelines to simulate the cervical vertebrae trabecular bone. This choice is reasonable given the need for anatomical detail in dosimetry but may limit generalizability since the cervical vertebrae structure might not fully represent other bone marrow sites.*

*The description could benefit from clarifying why the JM-103 model was chosen over other models or datasets, particularly those that could include bone tissues beyond the cervical vertebrae.*

**Response:** The following sentence was added to the Method section: “The cervical vertebrae were selected for modelling because they are simple in shape and easy to model.” The table of masses of bone tissues and the following sentence were added in the Discussion section: “The model does not reflect differences of mass of bone tissues according to location. The masses of bone tissues varies widely according to location in the bone as shown in Table 5.”

#### Discussion Section

*Despite noting the need for micro–computed tomgraphy–based models, the authors do not discuss how current limitations might impact dose estimation accuracy, especially for complex geometries in the trabecular bone. A clearer explanation of how simplified geometric assumptions may influence absorbed dose calculations would provide a balanced view of the model’s limitations.*

**Response:** The following sentence was added to the Discussion section: “The ratio of bone marrow and blood differs depending on the part of the bone, so the results obtained from the cervical vertebra model cannot be applied to the whole body. However, it is certainly necessary to perform dose assessment that takes into account the fine structure of the bone and the location of the HSCs.”

## Abbreviations

ICRP: International Commission on Radiological Protection
SAF: specific absorbed fraction
SI: International System of Units
